# Investigating cardiac stimulation limits of MRI gradient coils using electromagnetic and electrophysiological simulations in human and canine body models

**DOI:** 10.1002/mrm.28472

**Published:** 2020-08-19

**Authors:** Valerie Klein, Mathias Davids, Lothar R. Schad, Lawrence L. Wald, Bastien Guérin

**Affiliations:** 1 Computer Assisted Clinical Medicine, Medical Faculty Mannheim, Heidelberg University, Mannheim, Germany; 2 A. A. Martinos Center for Biomedical Imaging, Department of Radiology, Massachusetts General Hospital, Charlestown, Massachusetts, USA; 3 Harvard Medical School, Boston, Massachusetts, USA; 4 Harvard-MIT Division of Health Sciences and Technology, Cambridge, Massachusetts, USA

**Keywords:** cardiac stimulation, electromagnetic exposure safety, electromagnetic field simulation, heart model, magnetostimulation thresholds, MRI gradient field switching

## Abstract

**Purpose:**

Cardiac stimulation (CS) limits to gradient coil switching speed are difficult to measure in humans; instead, current regulatory guidelines (IEC 60601–2-33) are based on animal experiments and electric field–to-dB/dt conversion factors computed for a simple, homogeneous body model. We propose improvement to this methodology by using more detailed CS modeling based on realistic body models and electrophysiological models of excitable cardiac fibers.

**Methods:**

We compute electric fields induced by a solenoid, coplanar loops, and a commercial gradient coil in two human body models and a canine model. The canine simulations mimic previously published experiments. We generate realistic fiber topologies for the cardiac Purkinje and ventricular muscle fiber networks using rule-based algorithms, and evaluate CS thresholds using validated electrodynamic models of these fibers.

**Results:**

We were able to reproduce the average measured canine CS thresholds within 5%. In all simulations, the Purkinje fibers were stimulated before the ventricular fibers, and therefore set the effective CS threshold. For the investigated gradient coil, simulated CS thresholds for the x-, y-, and z-axis were at least one order of magnitude greater than the International Electrotechnical Commission limit.

**Conclusion:**

We demonstrate an approach to simulate gradient-induced CS using a combination of electromagnetic and electrophysiological modeling. Pending additional validation, these simulations could guide the assessment of CS limits to MRI gradient coil switching speed. Such an approach may lead to less conservative, but still safe, operation limits, enabling the use of the maximum gradient amplitude versus slew rate parameter space of recent, powerful gradient systems.

## 1 | INTRODUCTION

Fast sequences such as fast spin echo,^1^ EPI,^2^ balanced SSFP,^3^ or modern diffusion techniques^4^ require fast gradient switching, especially at high resolution. However, in modern gradient systems, it may not always be possible to reach the system’s maximum gradient switching rate and amplitude due to peripheral nerve stimulation (PNS)^5–8^ and/or cardiac stimulation (CS)^8–11^ limits. Unlike PNS,^12–14^ CS thresholds are difficult to measure in humans, and as a consequence, the CS limits described in the International Electrotechnical Commission (IEC) 60601–2-33 guidelines on MRI safety are based on a combination of animal experiments and simple electromagnetic (EM) simulations.^9,15,16^

Over the years, large numbers of animal CS experiments have been performed using electrodes.^10,16^ The resulting data typically report the smallest electrode current or current density (estimated as applied current divided by electrode area^16^) triggering CS, defined as the presence of an ectopic heartbeat on the electrocardiographic signal or as an action potential (AP) in the case of single-cell experiments. Reilly compiled these experimental data and rescaled the threshold current density to units of electric field (E-field) by assuming that the cardiac E-field threshold for the most sensitive population percentile is the same as the theoretical stimulation threshold of a 20-μm-diameter myelinated nerve (namely, 6.2 V/m).^16^ This assumption is based on a single experiment that reported a minimum current density threshold of approximately 0.1 mA/cm^2^ for CS at 60 Hz,^17^ which is close to the threshold simulated for a 20-μm peripheral nerve fiber with a neurodynamic model.^16^ The resulting E-field magnitude versus pulse duration curves can be fitted to exponential^18^ or hyperbolic strength-duration models,^19^ yielding an estimate of the characteristic stimulation time constant of cardiac tissue (~3 ms^16^). Finally, Reilly converted these E-field curves to practical dB/dt limits by calculating the E-field induced in an ellipsoidal, homogeneous body model exposed to a magnetic field that is uniform over the body model’s cross section.^9,16^ This procedure relies on several assumptions, and consequently, it is not clear whether the resulting CS limits are accurate. Nevertheless, the strength-duration parameters estimated by Reilly^9,16^ form the basis for the CS limit defined in the IEC 60601–2-33 safety guidelines.^15^ Specifically, the guidelines use a rheobase E-field value of 2 V/m (including an additional safety factor of 3) and a time constant of 3 ms:

(1) $$\text{E}<\frac{2\text{V}/\text{m}}{1-\exp\left(-\frac{{\text{τ}}_{\text{s,eff}}}{3\;\text{ms}}\right)},$$

where τ_s,eff_ is the effective pulse duration, defined as the ratio of the peak-to-peak field variation and the maximum time derivative of the gradient waveform in that period.^15^ For whole-body gradient systems, the more practical dB/dt limit is used^15^:

(2) $$\frac{\text{dB}}{\text{dt}}<\frac{20\text{T}/\text{s}}{1-\exp\left(-\frac{{\text{τ}}_{\text{s},\text{eff}}}{3\;\text{ms}}\right)},$$

which is based on the approximation that a 20-T/s B-field variation creates a 2-V/m E-field in the heart. The IEC 60601–2-33 standard also provides a PNS limit in the form of a hyperbolic (Lapicque^19^) expression as well as the option to use experimental threshold data. The parameters of that expression (first level controlled the operating mode, valid for whole-body gradients) are such that the IEC PNS and cardiac limits have a common asymptotic value of 20 T/s at long rise times.^15^ In practice, the PNS response of commercial MRI coils is measured in a cohort of healthy human subjects, however, which is more accurate and system-specific than the general IEC formula, so this expression is rarely used. For some high-performance gradient systems, the regulatory CS limit can be lower than the experimental PNS limit for long rise times. For the Massachusetts General Hospital–University of California, Los Angeles (MGH-UCLA) Connectome scanner^14^ (maximum gradient amplitude G_max_ = 300 mT/m and slew rate S_max_ = 200 T/m/s), for example, the experimentally measured PNS threshold surpasses the IEC cardiac limit at rise times greater than 1.5 ms and gradient amplitude greater than 100 mT/m (x + y + z axis combination^14^). This required implementation of a dedicated cardiac safety monitor to stop the acquisition if the CS limit is exceeded.^14^

As explained previously, the bulk of animal data on CS comes from electrode experiments, and there are very few experimental results available for magnetostimulation (ie, stimulation by E-fields generated by switching currents in an external inductor or coil, such as an MRI gradient coil). In the early 1990s, Yamaguchi, Mouchawar, and Nyenhuis performed magnetostimulation experiments on canines using coplanar loop and solenoid coils.^20–24^ Mouchawar and Nyenhuis correlated these results with E-fields simulated in a simple block thorax model of a dog with six tissue compartments,^25^ while Ragan et al expanded these simulations using a more detailed canine model.^26^ By combining the experimental canine results and the E-field simulations, the cardiac E-field threshold was found to range between 85 V/m and 100 V/m^25,26^ for the specific excitation waveforms used in the experiments. Other simulation studies investigated the E-field induced in the human heart by MRI gradient coils, but did not correlate those E-field values to CS thresholds.^27–29^ For example, Liu et al^29^ modeled MR gradient-induced CS by combining a heterogeneous voxel model of a man and an “electrical heart model” based on a mesh representation of the myocardium,^30,31^ with the goal of assessing the effect of possible CS on the cardiac rhythm. They did not simulate stimulation thresholds, but rather assumed that the heart was always excited at the locations of maximum induced E-field.^29^ The authors noted that all peak E-fields calculated in their study were lower than the E-field thresholds previously estimated from animal CS studies, which may indicate that the gradient coils they investigated were not in fact capable of inducing CS.^29^

In the present study, we introduce a modeling framework for the prediction of CS thresholds^32,33^ in EM body models by arbitrary coil wire patterns and waveforms. In contrast to the work of Liu et al, our approach includes realistic models of the topology of excitable fibers in the heart, which is important because the relative orientation of the E-field and the fiber is critical in predicting stimulation.^34^ For example, even a large E-field amplitude variation has little effect on a fiber if the E-field is oriented perpendicular to the fiber direction. In this work, we model cardiac Purkinje fibers and the myocardial fibers in the ventricles. Both fiber types play an important role in cardiac excitation and signal conduction and have different topological and electrophysiological properties. We validate our predictions by comparing the simulated CS thresholds with those measured in the canine studies by Mouchawar^22^ and Nyenhuis.^24^ While the main goal of our work is to predict CS thresholds of MRI gradients, to guide the establishment of safety guidelines, the proposed simulation tool may also be helpful in developing magnetic pacemaker and defibrillation devices.^11,35^

## 2 | METHODS

**FIGURE 1:**
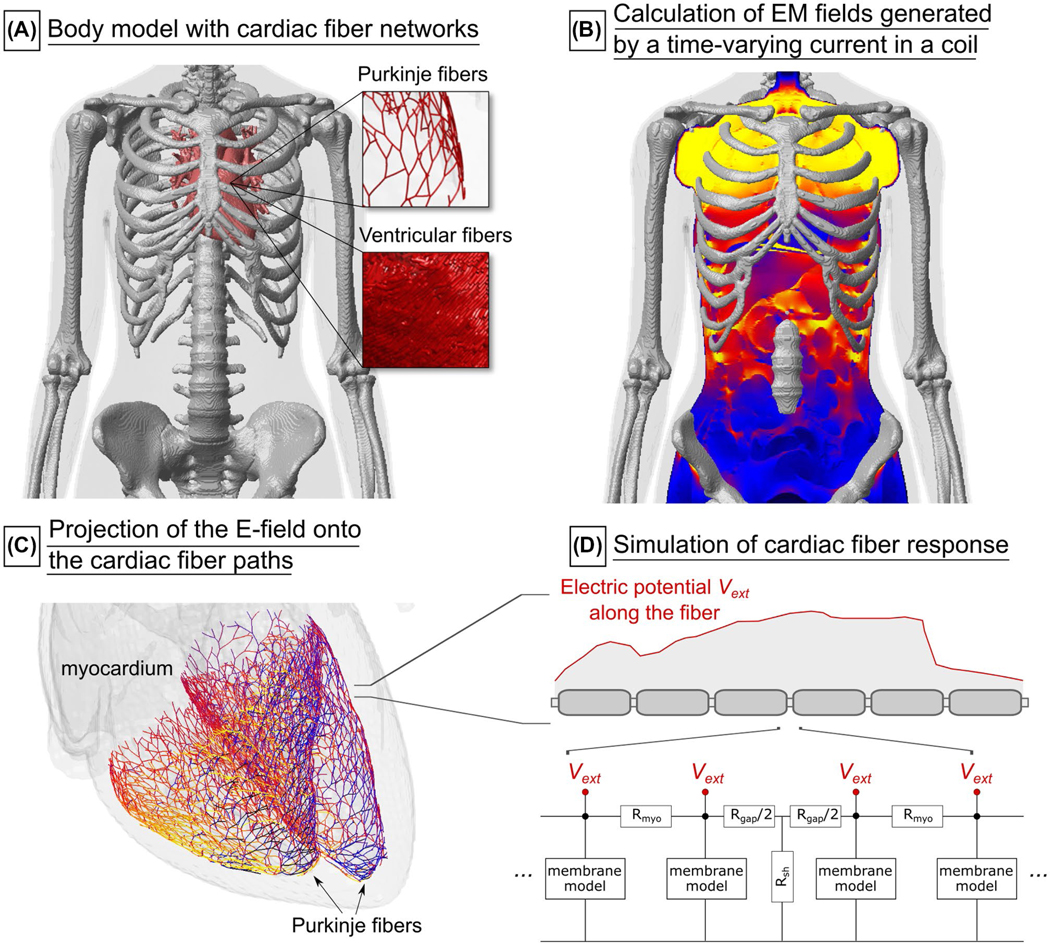
Overview of the simulation pipeline for the prediction of cardiac stimulation. A, Detailed body model (shown here are the bones, skin, and heart of the female human model) with added realistic networks of cardiac Purkinje and ventricular muscle fibers. B, Simulated electric fields (E-fields) induced by a time-varying current in a coil. C, The E-field is projected onto the cardiac fiber paths. D, The cardiac response to the extracellular electric potential is predicted using electrical-circuit models of Purkinje and ventricular muscle fibers. Abbreviation: EM, electromagnetic

The framework for modeling CS is an extension of our previous work on prediction and localization of PNS.^36–38^ Figure 1 gives an overview of the CS modeling workflow.

### 2.1 | Body models and cardiac fiber models

Our human body models are derived from the commercially available anatomical surface data of a male (weight 82 kg, height 176 cm) and female (weight 53 kg, height 163 cm) provided by Zygote (American Fork, UT). As described in previous publications, we processed the Zygote surfaces to make them suitable for EM field simulations, which involved regeneration of the surface models using a process of voxelization and remeshing, to make all meshes 2-manifold and watertight.^36,39^ We assigned electrical conductivity values to the different tissue classes using the IT’IS (Zurich, Switzerland) low-frequency database.^40^

For the canine simulations, we used the Human Monitoring Laboratory voxel model of an adolescent dog (14 kg, height to withers 44 cm, 20 tissue classes).^41^ The Human Monitoring Laboratory model only has a void space for the heart (no description of the atria and ventricles), which we remedied by inserting the atrial and ventricular surfaces of the female Zygote model using scaling, translation, and rotation. We generated five larger canine models from the Human Monitoring Laboratory model by applying geometrical scaling to all tissues/organs, including the heart. The scaling factor was chosen so the resulting models matched the minimum, mean, or maximum weight of the dogs used in the Mouchawar study^22^ (minimum = 17 kg, mean = 21.5 kg, maximum = 26 kg) and the Nyenhuis study^24^ (17 kg, 24.5 kg, and 32 kg).

**FIGURE 2:**
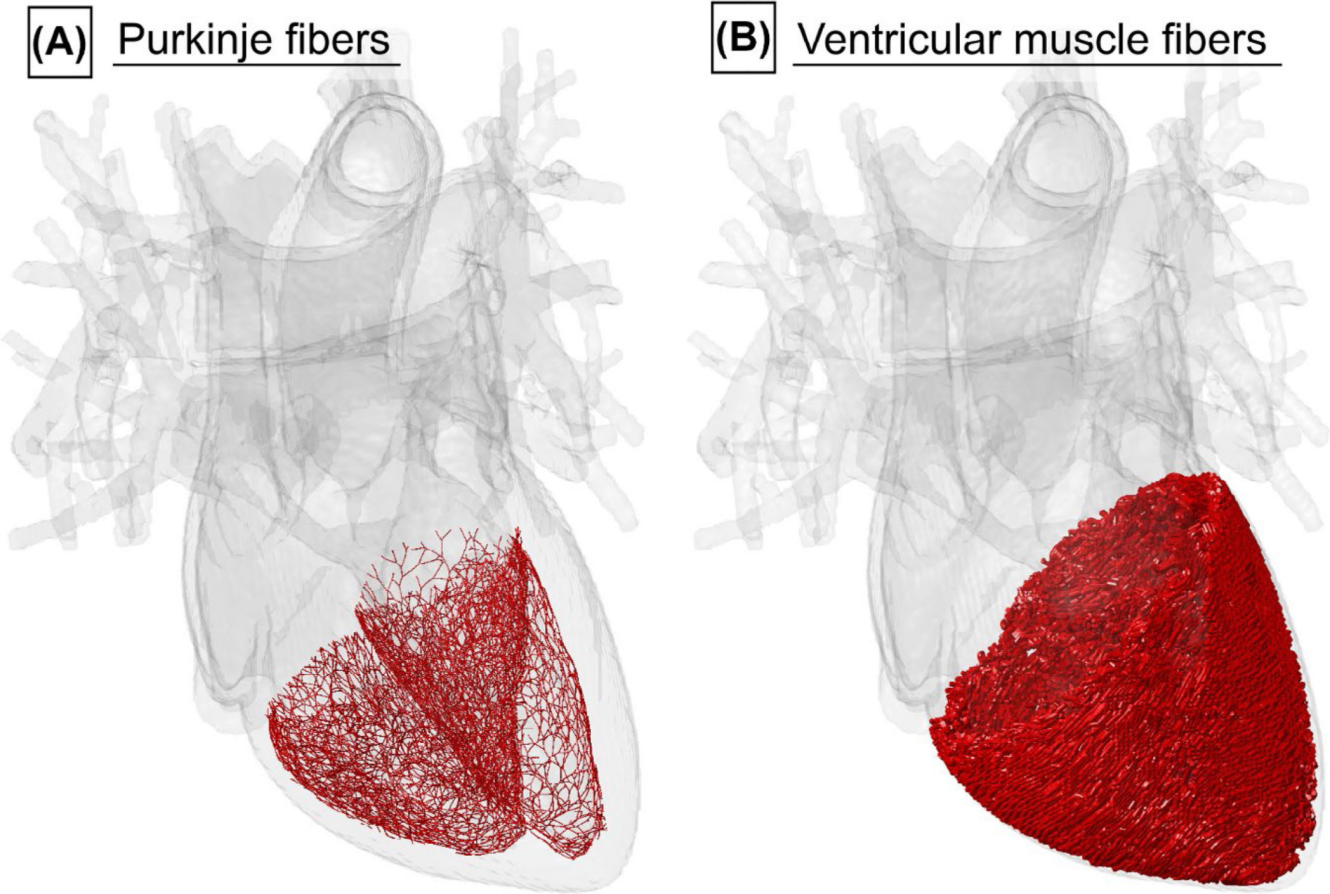
Surface model of the myocardium, the vena cava, aorta, and pulmonary arteries of the female body model. Superimposed in red are the Purkinje fibers (A) and the ventricular muscle fibers (B) that have been added to the model using rule-based modeling algorithms^42,43^

The body models contain a geometrical description of the myocardium, but not of the myocardial fibers involved in CS (Purkinje and ventricular muscle fibers). We generated Purkinje fiber networks in the left and right ventricles of the body models using a previously published rule-based growth algorithm specifically developed to mimic the structure of mammalian Purkinje networks.^42^ The algorithm starts with a single fiber segment, which is iteratively grown in a treelike fashion into “children” segments, the length of which is random and drawn from a Gaussian distribution (mean = 3 mm, SD = 0.2 mm). Each parent segment divides into two children, which is based on histology observations in sheep,^42^ and the angle between the children segments is random following a Gaussian distribution (mean = 60º, SD = 6º). Actual Purkinje fibers are not straight, which is modeled by dividing each fiber segment into subsegments, whose relative orientation controls the curvature of the overall segment. The order in which the parent segments are grown into children is randomized to avoid systematic bias in the network topology, and the growth stops when segments collide or when a segment grows too far away from the myocardial surface. Figure 2A shows a Purkinje fiber network we generated using this algorithm in the female human body model.

The second class of cardiac fibers that plays an important role in propagation of the cardiac excitation signal are the ventricular muscle fibers, which consist of interconnected cardiomyocytes and wrap around the left and right ventricles along helix trajectories. We generated ventricular fiber networks using the algorithm proposed by Bayer et al,^43^ which uses a tetrahedral mesh representation of the myocardium, on which two vector fields are defined: (1) the field of vectors pointing from the inner to the outer myocardial surface, and (2) the field of vectors pointing from the apex (ie, the tip of the heart) to the base (the superior surface of the heart muscle). Using these basic directions, fiber orientations are assigned to each mesh node based on observations from histology and DTI.^43^ Finally, streamlines representing the fiber paths are computed from the fiber orientation data. Figure 2B shows the resulting fiber paths that we generated in the ventricles of the female body model.

### 2.2 | E-field simulations

We calculated the E-fields induced by external coils (gradient, solenoid, and coplanar loops) in the human and canine models using the hexahedral finite-element-method magneto quasi-static solver of *Sim4Life* (Zurich MedTech, Switzerland) at 1-mm^3^ spatial resolution for a 1 A, f_0_ = 1 kHz sinusoidal coil current. We computed the E-field induced by an arbitrary current waveform and target B-field (or gradient) amplitude B_target_ in the region of interest using the following formula:

(3) $$\text{E}=\frac{\text{E}(1\;\text{A},1\;\text{kHz})}{2\pi f_{0}}\frac{{\text{B}}_{\text{target}\;}}{\varepsilon_{\text{B}}}\frac{\text{dW}(\text{t})}{\text{dt}},$$

where ε_B_ is the B-field efficiency of the coil at 1 A, and W(t) is the unitless waveform profile scaled between −1 and +1. Alternatively, the E-field can be scaled based on the desired gradient field amplitude, in which case ε_B_ is replaced by the gradient efficiency ε_G_. This simple linear scaling is valid in the quasi-static (low-frequency) regime.

**FIGURE 3:**
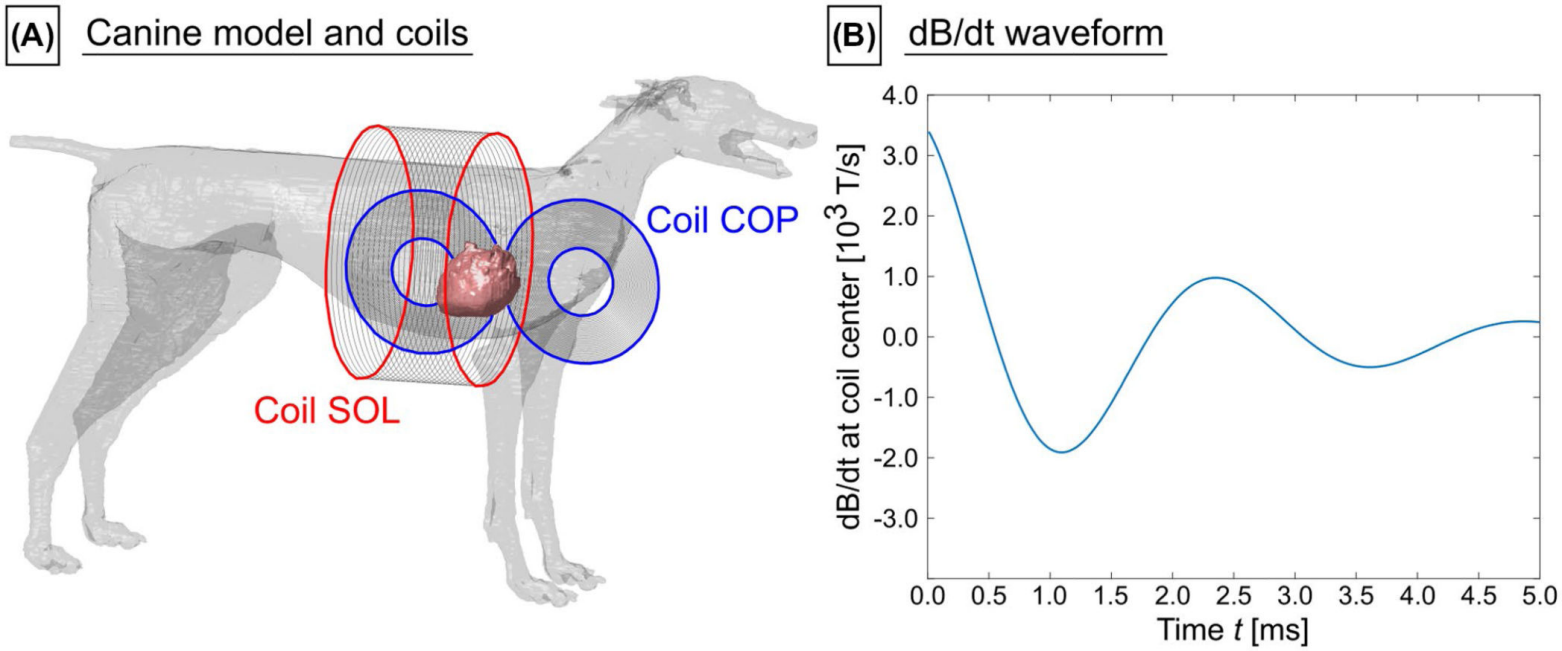
A, Canine body model (only skin and myocardium are shown) with a pair of coplanar coils (COP) and a solenoid coil (SOL). B, Simulated damped sinusoidal dB/dt waveform as generated in the experiments^22,24^ by discharging a capacitor into the coils. The time from onset to first zero crossing is 571 μs for COP, and 540 μs for SOL, respectively

For the canine simulations, we modeled the coils that were used in the experimental canine studies of Mouchawar and Nyenhuis^22,24^ (Figure 3A), namely: (1) a pair of coplanar coils placed on the left side of the canine’s torso (30 turns each, inner diameter = 7 cm, outer diameter = 17 cm, referred to as “COP”),^22^ and (2) a solenoid coil enclosing the canine’s torso (24 turns, diameter = 26 cm, length = 13 cm, referred to as “SOL”).^24^ In the experiments, a capacitor was discharged into the coils, resulting in a damped sinusoidal current waveform^22,24^ with duration from onset to first zero-crossing of the induced current equal to 571 μs for COP and 540 μs for SOL (this was reproduced in the simulations, Figure 3B). The maximum achievable B-field magnitude at coil center was approximately 5.9 T for COP (~1.7 T for SOL), and the maximum dB/dt at coil center was about 15.5 kT/s for COP (~4.8 kT/s for SOL).

For the human simulations, we modeled the actively shielded whole-body “Sonata” gradient coil (G_max_ = 40 mT/m, S_max_ = 200 T/m/s; Siemens, Erlangen, Germany) loaded with the male and female human body models head-first supine with the head at isocenter. We simulated gradient waveforms with sinusoidal and linear ramps (rise times between 0.1 ms and 5.0 ms, 500-μs flat-top duration, 10 bipolar pulses). In addition to the Sonata gradient, we modeled the y-axis gradient coil of the MGH-UCLA Connectome scanner^14^ (G_max_ = 300 mT/m, S_max_ = 200 T/m/s) loaded with the female body model with head at isocenter and driven with a gradient waveform with sinusoidal ramps.

### 2.3 | Electrophysiological simulations

The input of the Purkinje and ventricular fiber models is the spatiotemporal electric potential change along the fiber path, which is obtained by projection of the E-field onto the fiber trajectories and integration along those paths. Note that the E-field, and hence the potential change, is modulated in time by the coil current waveform W(t).

**FIGURE 4:**
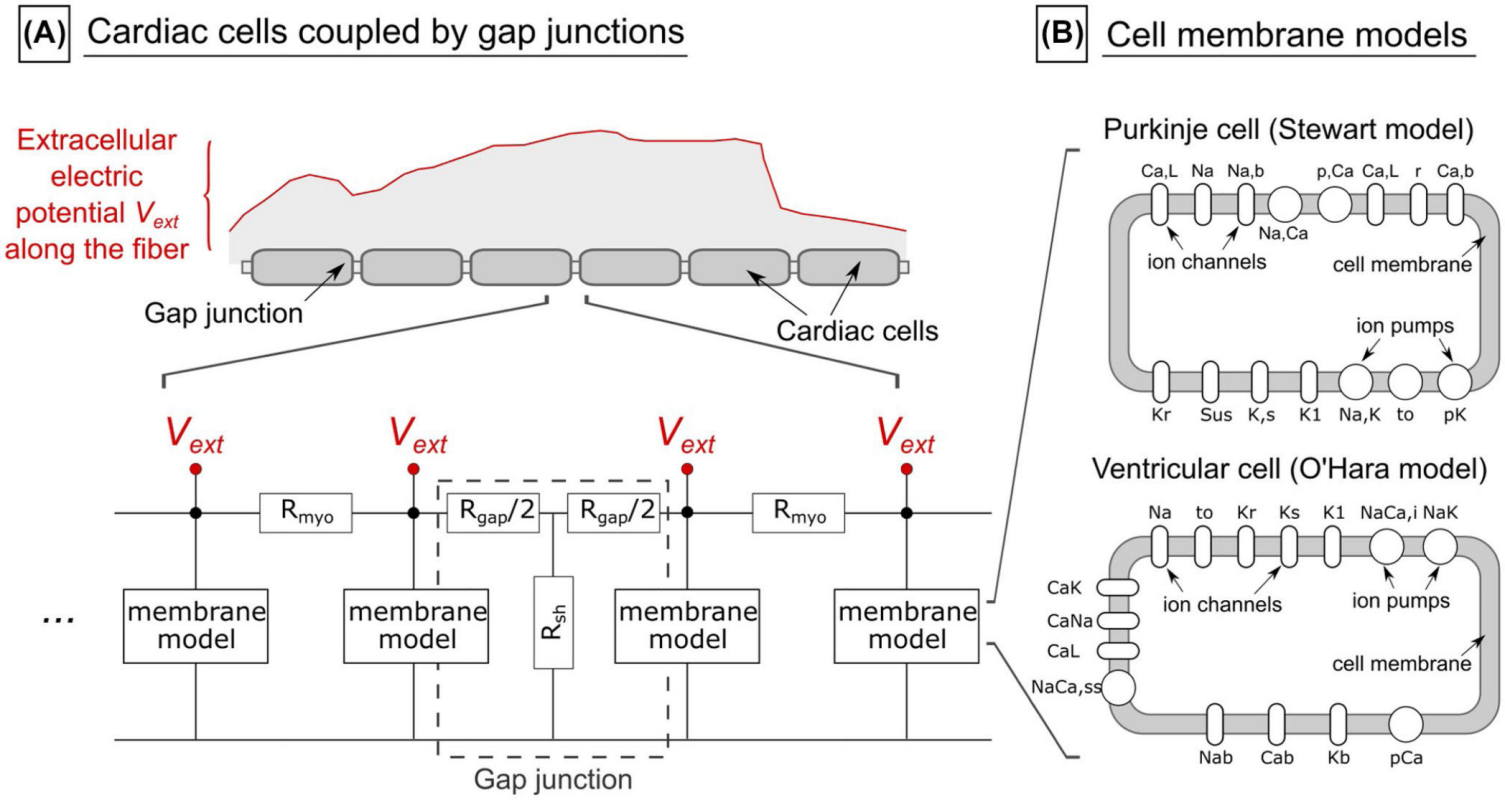
A, The cardiac fiber model consists of single cells coupled longitudinally by gap junctions, modeled as a resistive T-network.^44^ We assigned the axial gap junction resistance R_gap_ with 1 Ωcm^2^,^44^ the leakage resistance to extracellular space R_sh_ with 10^10^ kΩ,^44^ and the resistivity of the myoplasm R_myo_ with 162 Ωcm.^73^ B, Simplified depiction of the membrane models of Purkinje cells (Stewart model^45^) and ventricular muscle cells (O’Hara model^46^) showing the different ion channels and ion pumps

The generated Purkinje fiber and ventricular fiber networks consist of approximately 3000 and 7000 fiber segments, respectively. A single fiber segment consists of between 5 and about 1000 cylindrical cells, each 100 μm long. The cells are connected by gap junctions (80 Å in length) that are modeled as a resistive T-network, as described by Rudy and Quan^44^ (Figure 4). We modeled the membrane of individual cells in those fibers using the validated electrical-circuit Stewart model for Purkinje cells,^45^ and the O’Hara model for ventricular cardiomyocytes,^46^ as implemented in the CellML model repository.^47^ We assigned the largest physiological fiber diameters found in the human body for Purkinje fibers (80 μm) and ventricular fibers (15 μm),^48^ as this represents a conservative assessment of the stimulation thresholds (larger fibers are generally more excitable). We used the same fiber diameters for the human and canine simulations (for canines, Purkinje fiber diameters are known to be in the 20–200 μm range,^49,50^ while ventricular fibers are around 12 μm in diameter^51^).

The Purkinje and ventricular fiber models are described by a set of coupled differential equations modeling ionic current flow dynamics (calcium, sodium, and potassium) across the cell membrane, and signal propagation through the cells and gap junctions, which we solved using the Rush-Larsen algorithm.^52^ The Stewart model reproduces a peculiarity of the Purkinje fibers, namely, the ability to generate APs at a default rate (~50 beats per minute) even in the absence of an external stimulus. In this work, we define CS as the initiation of a single ectopic AP that does not result from the Purkinje auto-rhythmicity. We determined CS thresholds (ie, the smallest gradient amplitude triggering an AP) using a titration process, in which the amplitude of the coil current waveform (for a given rise time) is increased until an AP is observed. Note that an AP in a single fiber can lead to excitation of the entire myocardium.^53^

### 2.4 | Simulation of strength-duration curves

The response of excitable nerve and muscle tissue to an applied E-field is commonly evaluated using strength-duration curves,^54–57^ which quantify the threshold for AP generation in terms of the smallest amplitude of an applied unipolar rectangular E-field pulse as a function of the pulse duration. In most magneto-stimulation studies, such an E-field pulse is generated during the slew period of a trapezoidal coil current (and B-field) waveform.

We placed the human body models with the head at isocenter (head-first supine) in the gradient coil, and simulated coil current ramp durations (equal to the E-field pulse duration) between 0.2 ms and 10.0 ms. We increased the slope of the magnetic field ramp dB/dt until an ectopic AP was elicited in the cardiac fiber networks. We then determined the maximum E-field magnitude along the path of the stimulated fiber and plotted it as a function of pulse duration. The resulting simulated strength-duration curves were fitted with a nonlinear least-squares solver using two expressions that are used widely in the tissue stimulation literature:

- The hyperbolic Lapicque expression E(τ) = E_rheo_•(1 + t_chron_/τ),^19^ where τ is the pulse duration and t_chron_ is the chronaxie time, defined as the time at which the excitation threshold is twice the long-duration asymptote E_rheo_, the so-called “E-field rheobase”; and
- The exponential Blair expression E(τ) = E_rheo_ /(1 − exp (− τ/t_c_)).^18^ In this equation, τ is the pulse duration, E_rheo_ is the E-field rheobase, and t_c_ is the membrane time constant.

## 3 | RESULTS

### 3.1 | Cardiac stimulation threshold simulation in human models

**FIGURE 5:**
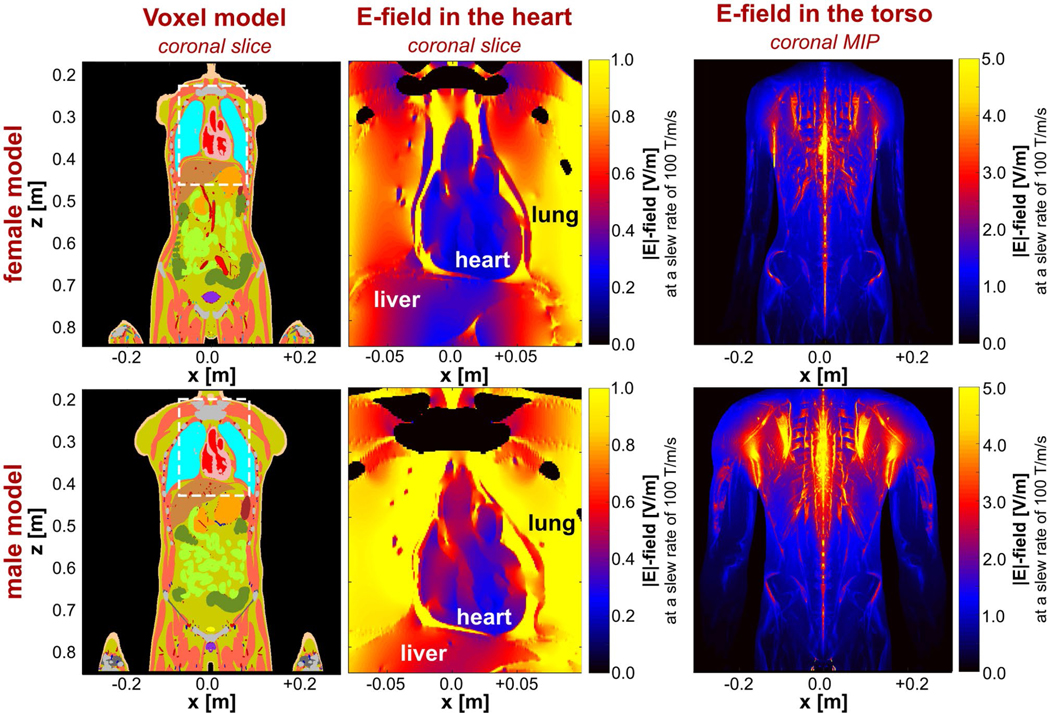
Coronal slices of the male and female voxel models (left column). The center column shows an enlarged section (dashed box in the left column) of the E-field induced in this slice. The right column shows E-field maps in the whole torso as maximum intensity projections (MIPs) of E-field values along the y-direction onto the xz-plane. All E-fields were induced by the Sonata gradient’s z-axis at a slew rate of 100 T/m/s. The E-field in the bones is set to zero for better visibility

The gradient efficiency of the Sonata coil was about 0.092 mT/m/A for all axes in our simulations, which is in agreement with the manufacturer’s specifications. Figure 5 shows the E-fields induced in the male and female human body models by the z-axis gradient coil driven with a 1-kHz sinusoidal current waveform producing a maximum slew rate of 100 T/m/s. The E-field in the heart is significantly lower than in the surrounding tissues: The 95th percentile E-field amplitude in the myocardium is 1.2 V/m for the male model (0.9 V/m for the female model), whereas the maximum E-field amplitude in the whole torso is 9.5 V/m for the male model (14.2 V/m for the female model).

**FIGURE 6:**
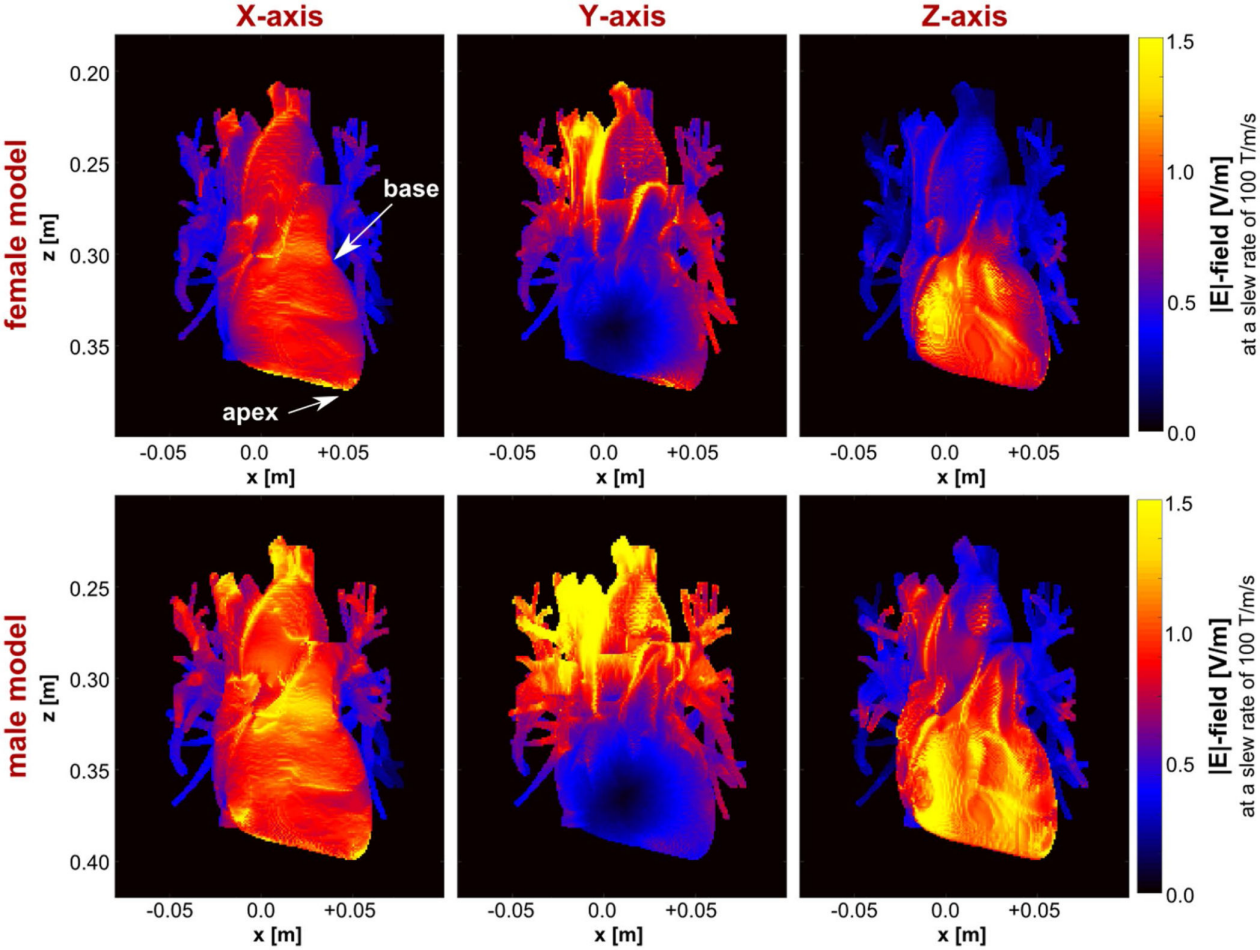
E-field (MIP) induced in the myocardium, the vena cava, aorta, and pulmonary arteries of the male and female body models (rows) by each gradient axis (columns) at a slew rate of 100 T/m/s. The E-field outside the heart is set to zero for better visibility

Figure 6 shows maximum intensity projections of the E-field induced in the heart by each gradient axis at a slew rate of 100 T/m/s. The E-field distributions are highly heterogeneous, with higher E-fields being induced in the male model. For both body models, the z-axis induces the highest E-field in the myocardium, followed by the x-axis, which induces particularly high E-fields around the apex. The y-axis induces high E-fields in the vena cava, aorta and pulmonary artery, but relatively low E-fields in the myocardium.

**FIGURE 7:**
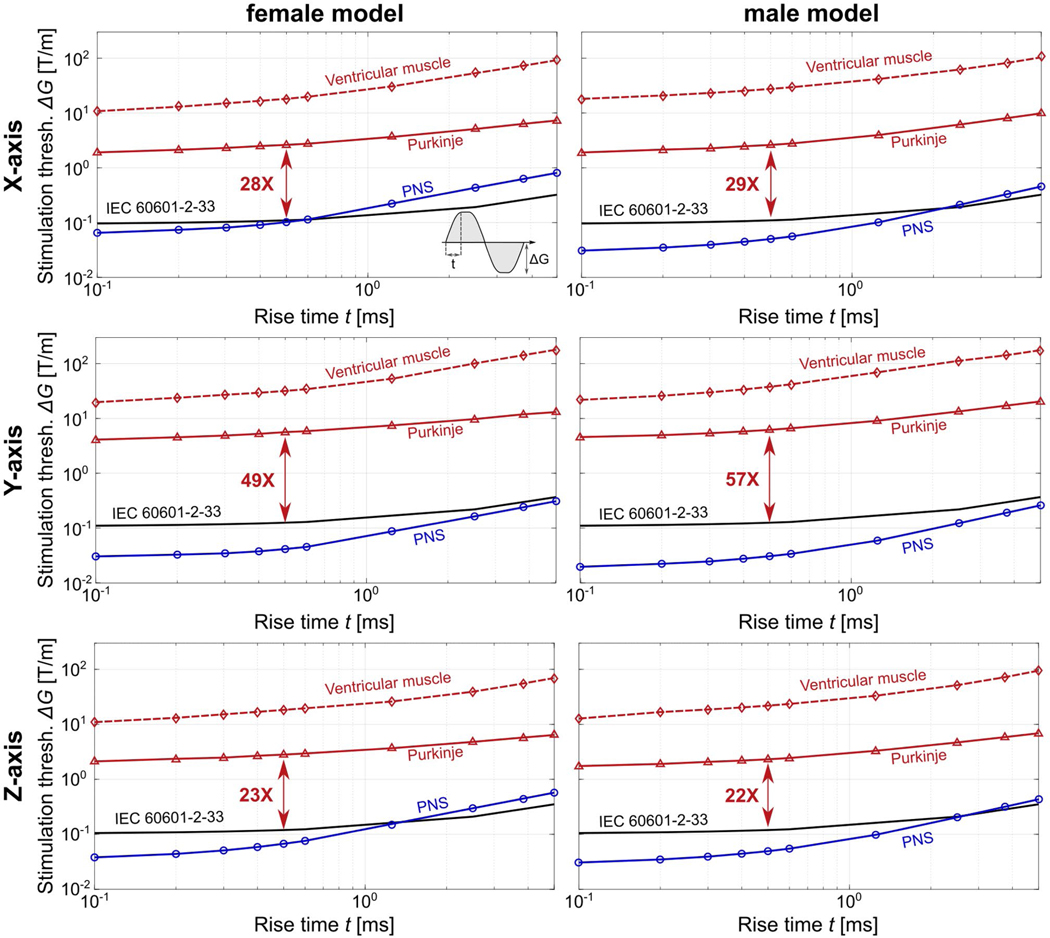
Stimulation thresholds of the Sonata gradient coil in terms of gradient amplitude *ΔG* as a function of the rise time *t* for a gradient waveform with sinusoidal ramps. Thresholds are plotted for all gradient axes (rows) and both human body models (columns). The cardiac stimulation (CS) thresholds are plotted in red (Purkinje fibers as solid lines, ventricular muscle fibers as dashed lines), simulated peripheral nerve stimulation (PNS) thresholds are plotted in blue, and International Electrotechnical Commission (IEC) 60601–2-33 cardiac safety limits are shown in black

Figure 7 shows the simulated CS thresholds of the male and female body models for the three axes of the Sonata gradient driven with the gradient waveform with sinusoidal ramps (thresholds for the waveform with linear ramps are shown in Supporting Information Figure S1). We show the stimulation thresholds for the Purkinje and ventricular fibers (red triangles and diamonds, respectively), the IEC 60601–2-33 safety limits (black), and the simulated PNS thresholds (blue, as calculated in an earlier publication^37^). For all gradient axes and both body models, the Purkinje fibers are 3 to 20 times more sensitive to stimulation than the ventricular fibers. The stimulation loci of the Purkinje fibers approximately coincide with the locations of maximum E-field magnitude (see Supporting Information Figure S2). More specifically, the simulated stimulation thresholds correlate well with the inverse of the second spatial derivative of the electric potential along the fibers (correlation coefficient ~0.964, Supporting Information Figure S3), which is in agreement with previous experiments and simulations.^34^ We assessed the impact of the exact topology of the Purkinje fiber network on the threshold values by generating four additional semirandom Purkinje networks for the female model: The resulting threshold variability ranged from 30% at short rise times up to 75% at long rise times (Supporting Information Figure S4). We used the Purkinje fiber network with the lowest thresholds for all simulations in this work to obtain a conservative threshold estimate. Finally, we observed that the ratio between the simulated Purkinje fiber and PNS thresholds depends on rise time: At short rise times (<0.5 ms), the CS thresholds are at least two orders of magnitude greater than the PNS thresholds, whereas at long rise times (>2.5 ms), this ratio decreases to approximately one order of magnitude. In all simulations, the CS thresholds simulated for the Sonata gradient are significantly higher than the IEC cardiac safety limits (≥22 times higher at t = 0.5 ms). We found an even greater margin between simulated CS thresholds and IEC cardiac safety limit for the y-axis of the Connectome gradient (80 times higher CS threshold at t = 0.5 ms; Supporting Information Figure S5).

**FIGURE 8:**
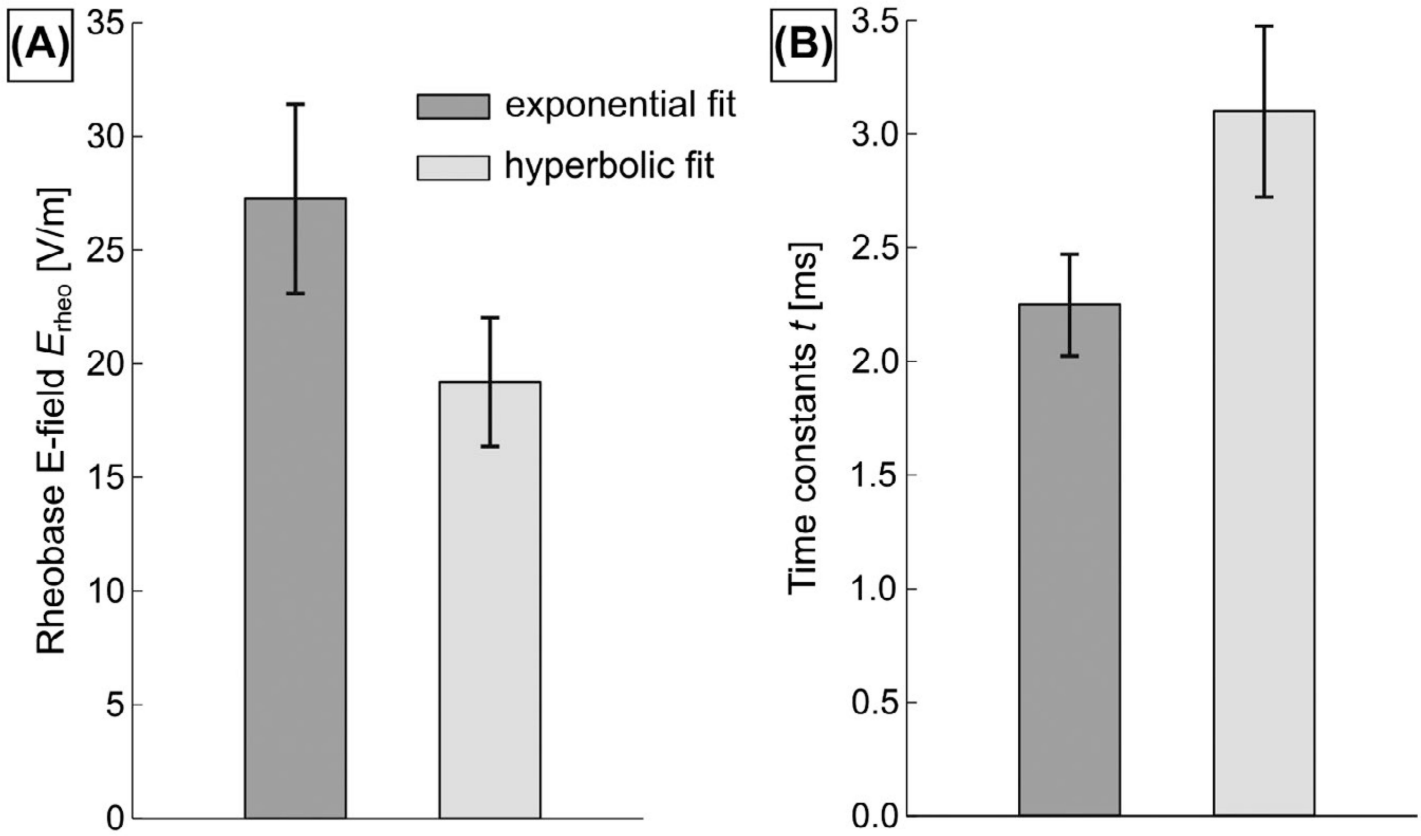
Electric-field rheobase (A) and time constants (B) obtained from fitting exponential and hyperbolic strength-duration models to the “E-field threshold” versus “pulse duration” curves for the two human models and the three gradient axes. The fit results are shown in terms of mean ± SD of all six simulations (see individual fit curves in Supporting Information Figure S6)

Figure 8 shows the exponential and hyperbolic strength-duration fit parameters (mean ± SD for all axes of the Sonata gradient and the two human body models), while Supporting Information Figure S6 shows the individual fit curves. In our simulations, the hyperbolic strength-duration expression^19^ describes the E-field thresholds better (RMS error ≤ 4.4 V/m) than the exponential expression^18^ (RMS error ≤ 7.1 V/m).

### 3.2 | Cardiac stimulation threshold simulation in canine models

**FIGURE 9:**
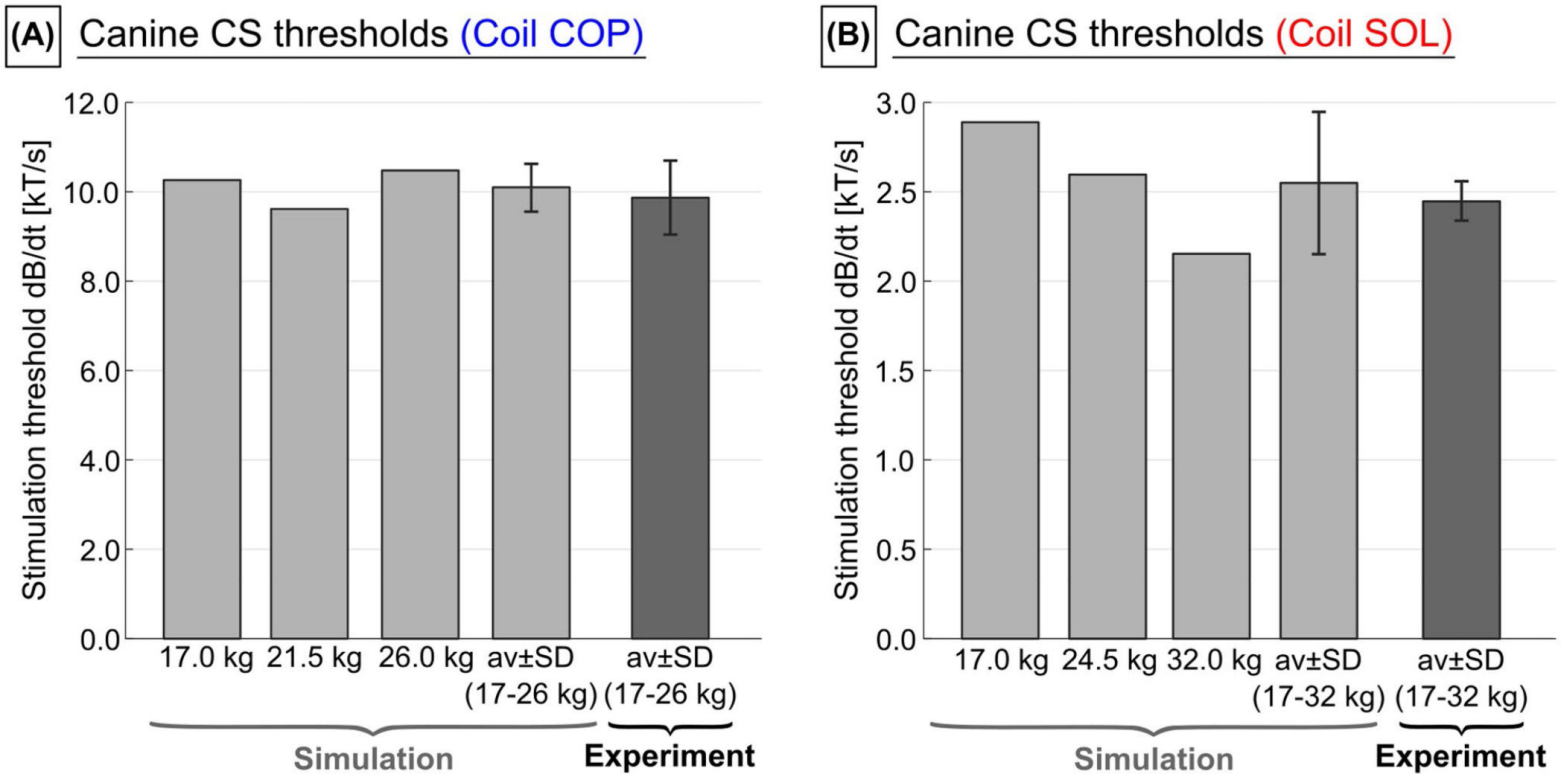
Simulated and experimental CS thresholds of the dogs for the coplanar coils (COP) and the solenoid coil (SOL) in terms of peak dB/dt amplitude. All thresholds are given for an equivalent rectangular waveform of 571 μs (COP) and 540 μs (SOL). The simulated thresholds correspond to the Purkinje fiber thresholds (thresholds of the ventricular muscle fibers were approximately 6-fold higher)

Supporting Information Figure S7 shows the E-fields induced in the 17-kg canine body model. The 95th percentile E-field amplitude in the myocardium is (6.5 ± 0.6) V/m for COP, and (20.3 ± 1.5) V/m for SOL (mean ± SD of the respective canine models). Figure 9 summarizes the simulated and experimental canine CS thresholds. All thresholds are scaled to an equivalent rectangular dB/dt waveform of 571 μs (COP) and 540 μs duration (SOL), respectively, as done by Mouchawar et al.^22^ As in the human body models, the Purkinje fibers of the canine models are about 6-fold more sensitive to stimulation than the ventricular fibers, and hence set the effective threshold. We found good agreement between the average simulated and experimental thresholds for both coils, with the simulated thresholds exceeding the experimental thresholds by about 2% (COP) and 4% (SOL).

## 4 | DISCUSSION

We have developed a modeling framework to predict CS in humans and animals by E-fields induced by external AC-driven coils, which extends our previously validated PNS model.^36–38^

### 4.1 | Validation

We validated our modeling against two canine studies conducted by Mouchawar et al^22^ and Nyenhuis et al^24^ in 1992. Mouchawar et al studied CS in 11 dogs using coplanar loop coils (COP), while Nyenhuis et al used a solenoid coil (SOL) and a cohort of 12 dogs. These authors were also interested in the study of MRI gradient safety, but were not able to achieve CS in canines using a small 26-cm diameter, 55-cm-long gradient coil driven by a large capacitor.^23^ The publications with the solenoid and coplanar coils reported some of the experimental details that we attempted to reproduce in our simulations. However, it is difficult to replicate the exact anatomy of the dogs used in these experiments. Instead, we used a Doberman body model (Human Monitoring Laboratory model^41^) that we scaled geometrically to reproduce the weights of the dogs from the experiments.

Despite this caveat, we found excellent agreement between the average simulated and measured thresholds (< 5% error). For coil COP, the SD predicted by our model was 5%, while it was 8% in the experiments.^22^ For coil SOL, the SD of thresholds predicted by our model was 14% (with smaller thresholds for larger dogs), whereas it was only 5% in the experiments.^24^ In other words, our simulations reproduced the average stimulation thresholds for both coils, but only the threshold variability for coil COP. One reason for this may be that in the SOL study, CS was only achieved in 5 of the 12 dogs,^24^ and although the size and weight of the dogs with successful stimulation were not reported, it is likely that they all were among the larger ones. This may explain why the experimental threshold variability is smaller than in the simulations, which covered the whole weight range of all 12 dogs.

The damped sinusoidal waveform used for validation of our modeling in comparison with the experimental results is not representative of the traditional waveforms used in MRI. In an effort to translate these results to MRI, we modeled the COP and SOL coils loaded with the 17-kg canine model and excited using a trapezoidal waveform (10 lobes) with 0.5-ms rise time, which mimics the duration of the first-quarter period of the damped sine. For both coils, the cardiac thresholds were about 20% smaller than with the damped sinusoidal waveform.

### 4.2 | Simulated CS thresholds versus IEC 60601–2-33 safety guideline

The IEC 60601–2-33 CS safety limit^15^ is based on the exponential strength-duration curve derived by Reilly from a large number of animal electrode experiments.^16^ This curve is characterized by an E-field rheobase of 6.2 V/m and a time constant of 3 ms^15,16^ (the rheobase in the IEC guidelines contains an additional safety factor of 3, and is therefore 2 V/m). To translate these parameters into dB/dt limits, an E-field-to-dB/dt conversion factor of 10 (T/s)*(V/m)^−1^ was derived from simple EM field simulations modeling the body as an ellipsoid with homogeneous conductivity exposed to a magnetic field that is uniform over the body model cross section.^9,16^

In our simulations, we found that the IEC CS limit of the Sonata gradient coil was at least 20 times more conservative than the CS thresholds predicted for the Purkinje fibers (the most sensitive fibers in our model). The simulated ventricular muscle fiber thresholds were greater than the Purkinje thresholds, possibly owing to the smaller fiber diameter, and different morphology and membrane dynamics. Moreover, the simulated CS thresholds were about 10-fold (t > 2.5 ms) to 100-fold (t < 0.5 ms) greater than the PNS thresholds for all gradient axes. In our view, there are two main reasons for this. First, the heart is located deeper in the body than the peripheral nerves, and is therefore more shielded from the induced E-fields. Second, it is known that cardiac fibers have a longer time constant and greater rheobase (in the parlance of strength-duration curves) than peripheral nerve fibers.^10^ For most MRI scanners and pulses, the PNS threshold is the limiting factor compared with the IEC cardiac limit. In some gradients, however, such as the Connectome y-axis (Supporting Information Figure S5), the cardiac level is met first for long rise-time pulses (eg, >2.5 ms for the Connectome y-axis). This regime is met in long, high gradient-amplitude diffusion pulses. Note that our simulation of the Connectome y-axis coil shows qualitatively different behavior: The cardiac threshold is well above the PNS thresholds for all rise times studied. Because of the large difference between PNS and cardiac thresholds simulated with our framework (pending more validation), it is unlikely that cardiac thresholds become a concern even for modern, powerful coils with increased slew rate and amplitude capabilities.

Comparing our simulations of the y-axis of the Sonata gradient (Figure 7) and the Connectome gradient (Supporting Information Figure S5), we see that coils with similar overall coil winding topologies can yield widely different cardiac thresholds. Although both coils are symmetric and do not have funnel, flanges or folds, their different coil diameter (~70 cm for the Sonata, ~63 cm for the Connectome) and diameter of the spherical volume of linearity (40 cm for the Sonata, 20 cm for the Connectome) is sufficient to generate different cardiac thresholds. The Connectome y-axis CS threshold is about 67% greater than that of the Sonata y-axis at t = 0.5 ms. This may reflect the fact that for the Sonata gradient, the peak B-fields (and E-fields) are placed in the cardiac region when the head is at isocenter, whereas for the shorter Connectome coil, the peak B-field occurs slightly higher in the chest. We plan to use our modeling tool to gain more intuition on the effect of coil-design parameters on cardiac thresholds, as, unlike PNS, this is difficult to gain from experimental data. Our modeling framework could prove useful in gaining insight into the impact of different design features such as noncylindrical coil formers, use of multiple layers, and asymmetrical designs. Eventually, MRI gradient coils could be optimized to have inherently high cardiac safety limits, such as by reducing the E-field induced by the coil in the heart. This could be done in a similar fashion as for PNS, for which we have recently demonstrated that inclusion of a linearized PNS metric^58^ during the gradient coil design process can result in coils with significantly greater PNS thresholds.^59^

### 4.3 | Simulation of strength-duration curves

Electric-field strength-duration curves are widely used to evaluate nerve and muscle stimulation without the need to use advanced electrophysiological models such as the ones used in this work. However, there are indications that such curves are not universal. For example, Irnich reported values for the E-field rheobase (hyperbolic formulation) between 20 V/m and 147 V/m based on a compilation of 17 experimental studies on humans and animals.^10^ Reilly compiled another set of 21 experiments in different animal species (eg, dogs, sheep, rabbits, guinea pigs) using varying electrode configurations and stimulation loci. He analyzed the data using the exponential formulation and reported time-constant values between 0.2 ms and 7.7 ms.^16^ In other words, neither the E-field rheobase nor the time constant appear to be conserved across species, stimulation strategies, and stimulation locations. There are at least two reasons for this: First, the E-field estimation in the experiments is often based on simplistic assumptions (such as simple geometrical tissue shapes with homogeneous conductivity^22^). Second, the time constants are known to depend strongly on body temperature,^60^ fitting method,^55^ and electrode size.^16^

In the past few decades, a lot of work has been invested in the development of detailed models of cardiac electrophysiology, including realistic models of Purkinje and myocardial fibers.^61,62^ A central motivation for our work is that leveraging such models can lead to more accurate predictions of CS thresholds. We simulated strength-duration curves in the male and female body models (Sonata gradient coil) to compare our approach with previous threshold estimations and found, in the hyperbolic formulation, an average rheobase value of E_rheo,hyp_ = 19.0 ± 2.8 V/m, which is at the lower end of the experimental range (20 V/m to 147 V/m^10^). The simulated chronaxie was slightly greater (3.1 ± 0.4 ms) than the average experimental value suggested by Irnich (2.0 ms^10^). In the exponential formulation, we found an average E-field rheobase E_rheo,exp_ of 27.3 ± 4.2 V/m, which is significantly greater than the median E_rheo,exp_ of 12 V/m proposed by Reilly (for the most sensitive population percentile, Reilly proposed to use E_rheo,exp_ = 6.2 V/m,^9,16^ a value similar to the rheobase of large peripheral nerves^16^). The average simulated time constant is 2.2 ± 0.2 ms, which falls within the relatively large experimental range (0.2 ms to 7.7 ms^16^). In other words, our simulations broadly agree with the previously published literature values.

### 4.4 | Limitations and outlook

Potential improvements of our CS model include refinement of the canine heart anatomy using a more detailed segmentation of the myocardium. Our cardiac model only includes modeling of the Purkinje and ventricular fibers, as these make up the majority of fibers in the heart. Therefore, a limitation of our simulations is the absence of other classes of excitable tissue such as the atrial fibers and the sinoatrial and atrioventricular node cells. These are known to have different stimulation thresholds due to differing underlying membrane dynamics and morphology. For example, electrode stimulation experiments have shown that atrial fibers have a similar or greater stimulation threshold than ventricular fibers,^63,64^ and therefore are not expected to be the first to be stimulated. Less data are available about the sinoatrial and atrioventricular node cells, but experiments indicate that these cells also have greater thresholds than those of ventricular tissue.^65^ Nonetheless, we plan to include those additional fibers and cells^66–68^ in future versions of our model, to verify that these do not lead to changes in the predicted thresholds. In addition, we also plan to investigate possible stimulation of the nerves of the autonomous nervous system, such as the vagal nerve, which regulates the cardiac rhythm.^69^ Moreover, we plan to simulate more body positions and coil geometries, such as MRI head gradients, for which the IEC standard does not provide CS limits in terms of dB/dt.

Future simulations should include more body models including obese adults, pregnant women, and children of various ages, to better reflect a diverse population. Similarly to our previous PNS sensitivity analysis,^70^ it would also be informative to assess the effect of different model parameters such as body size, weight, shape, and electrical tissue conductivities on the CS thresholds. This appears to be important, as we have found in this study that even mild changes of the Purkinje fiber paths can have a significant effect on the stimulation thresholds (Supporting Information Figure S4). Indeed, the five semirandom Purkinje networks modeled in this work had, in the same female body model, a 30% to 75% variability in CS thresholds (greater variability at long rise times). Note that this significant physiological variability may partly explain the large variation of CS thresholds measured in vivo.^16^

The validation work presented here is encouraging, but probably insufficient, as it is limited to a single nonhuman species, dogs, which are not the most prevalent cardiac model. Furthermore, it was difficult to exactly reproduce the experimental setups of the 1992 canine studies. Therefore, our next step will be to further validate our simulations using CS threshold measurements in healthy pigs,^71^ which have emerged as the primary animal model for the human cardiovascular system.^72^ Creation of accurate EM body models based on the individual animal’s MRI images will help to remove the unavoidable simulation/measurement mismatches of the present study, and will therefore allow a more rigorous test of the validity of our CS predictions. Data from the MGH-UCLA Connectome scanner shows that the experimental PNS thresholds and IEC cardiac limit cross at relatively long pulse durations (rise time > 1.5 ms^14^). Our simulations of the Connectome y-axis gradient do not show this crossover, but rather indicate a large margin between CS thresholds and PNS thresholds. The accuracy of our modeling is therefore likely important in this regime, which we plan to study carefully in the upcoming porcine experiments.

## 5 | CONCLUSIONS

We introduce a modeling framework for the prediction of CS induced by external EM coils (magnetostimulation). This framework is an extension of our previous work on PNS modeling and includes realistic models of the cardiac Purkinje and ventricular muscle fiber topology and electrophysiology. Using this simulation approach, we were able to reproduce previously published experimental canine CS thresholds within 5%. Simulations of a commercial MRI gradient coil indicate that human CS thresholds for this coil are more than an order of magnitude greater than the IEC 60601–2-33 cardiac safety limit. With additional validation, we believe that our simulations may become a valuable tool to study cardiac magneto-stimulation in humans. Knowledge about CS thresholds and loci may help establish adequate safety limits for the exposure of the human body to time-varying magnetic gradient fields, to guarantee safe MRI operation while fully exploiting the performance of the imaging system. Furthermore, our CS tool could become useful for studying and optimizing therapeutic devices such as magnetic cardiac pacemakers.

## Supplementary Material

fig S1-S7 **FIGURE S1** Stimulation thresholds of the Sonata gradient coil in terms of threshold gradient amplitude ΔG as a function of the rise time *t* for a gradient waveform with linear ramps. The thresholds are plotted as in Figure 7. The trapezoidal gradient waveform led to overall higher stimulation thresholds than the sinusoidal waveform
**FIGURE S2** Top: E-field magnitude sampled along the Purkinje fibers of the female body model for the three gradient axes. The E-field was scaled to an equivalent slew rate of 100 T/m/s. Bottom: Stimulation loci of the 50 most sensitive fibers in the Purkinje network (larger spheres correspond to lower thresholds)
**FIGURE S3** Stimulation thresholds of the Purkinje fibers (female body model, gradient Z-axis) as a function of the inverse second spatial derivative of the electric potential along the respective fiber
**FIGURE S4** Stimulation thresholds (female body model, gradient Z-axis) computed for five different Purkinje fiber networks generated with the random Purkinje growth algorithm. Stimulation thresholds were modeled for a gradient waveform with sinusoidal ramps and rise times between 0.2 ms and 5.0 ms. The threshold variability across the different fiber networks ranged from 30% at short rise times up to 75% at long rise times
**FIGURE S5** Stimulation thresholds of the Massachusetts General Hospital–University of California, Los Angeles Connectome gradient coil y-axis in terms of gradient amplitude ΔG as a function of the rise time *t*. The cardiac stimulation (CS) thresholds (red) were simulated for a gradient waveform with sinusoidal ramps (500 μs flat-top duration, 10 bipolar pulses) in the female body model with the head placed at isocenter. The International Electrotechnical Commission (IEC) 60601–2-33 cardiac safety limits are shown in black. The PNS thresholds (blue) were previously measured in an experimental study in healthy volunteers for rise times between 0.1 ms and 0.8 ms,^14^ and were extrapolated linearly for higher rise times
**FIGURE S6** Strength-duration fit curves (hyperbolic Lapicque expression in solid blue, exponential Blair expression in dashed green) in terms of threshold E-field magnitude along the most sensitive cardiac fiber as a function of E-field pulse duration for the male and female body models (rows) and the three gradient axes (columns)
**FIGURE S7** Transverse slices of the voxel model and the E-field maps induced in the 17-kg canine body model by the coplanar coils (COPs) and the solenoid coil (SOL) at a peak magnetic field switching rate of dB/dt = 1000 T/s at coil center. The E-field in the bones is set to zero
