# Supplementary material for: Investigating cardiac stimulation limits of MRI gradient coils using electromagnetic and electrophysiological simulations in human and canine body models
Source: Magn Reson Med. Author manuscript; Available in PMC 2021 Feb 1. (PMC7722025; doi:10.1002/mrm.28472)
Supplement: fig S1-S7 — FIGURE S1 Stimulation thresholds of the Sonata gradient coil in terms of threshold gradient amplitude ΔG as a function of the rise time t for a gradient waveform with linear ramps. The thresholds are plotted as in Figure 7. The trapezoidal gradient waveform led to overall higher stimulation thresholds than the sinusoidal waveform FIGURE S2 Top: E-field magnitude sampled along the Purkinje fibers of the female body model for the three gradient axes. The E-field was scaled to an equivalent slew rate of 100 T/m/s. Bottom: Stimulation loci of the 50 most sensitive fibers in the Purkinje network (larger spheres correspond to lower thresholds) FIGURE S3 Stimulation thresholds of the Purkinje fibers (female body model, gradient Z-axis) as a function of the inverse second spatial derivative of the electric potential along the respective fiber FIGURE S4 Stimulation thresholds (female body model, gradient Z-axis) computed for five different Purkinje fiber networks generated with the random Purkinje growth algorithm. Stimulation thresholds were modeled for a gradient waveform with sinusoidal ramps and rise times between 0.2 ms and 5.0 ms. The threshold variability across the different fiber networks ranged from 30% at short rise times up to 75% at long rise times FIGURE S5 Stimulation thresholds of the Massachusetts General Hospital–University of California, Los Angeles Connectome gradient coil y-axis in terms of gradient amplitude ΔG as a function of the rise time t. The cardiac stimulation (CS) thresholds (red) were simulated for a gradient waveform with sinusoidal ramps (500 μs flat-top duration, 10 bipolar pulses) in the female body model with the head placed at isocenter. The International Electrotechnical Commission (IEC) 60601–2-33 cardiac safety limits are shown in black. The PNS thresholds (blue) were previously measured in an experimental study in healthy volunteers for rise times between 0.1 ms and 0.8 ms,14 and were extrapolated linearly for higher rise times FIGUR [file NIHMS1631195-supplement-fig_S1-S7.docx]

**Investigating cardiac stimulation limits of MRI gradient coils using electromagnetic and electrophysiological simulations in human and canine body models**

Valerie Klein, Mathias Davids, Lothar R. Schad, Lawrence L. Wald, Bastien Guérin

**Supporting Information Figures**


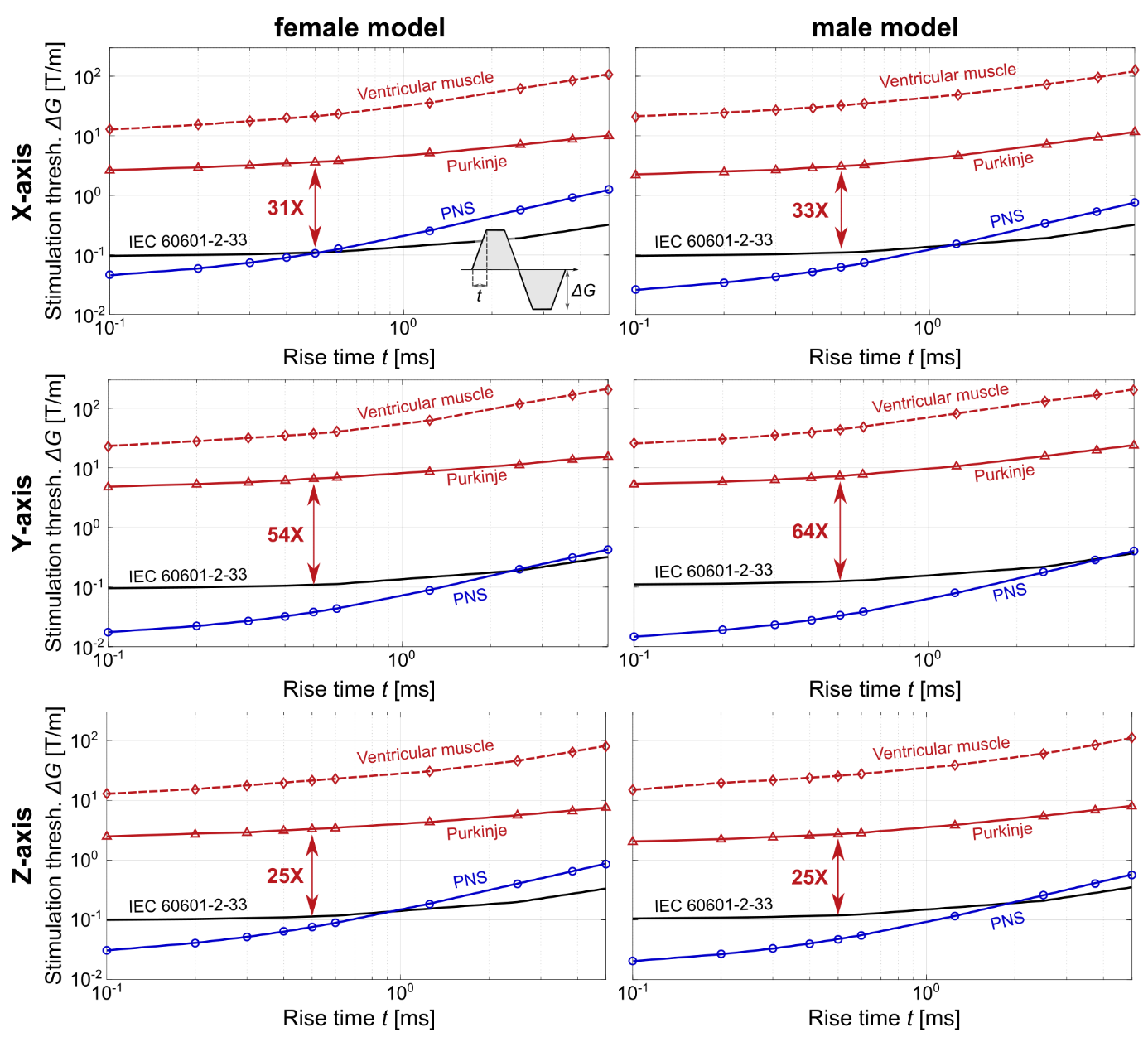


**Figure S1:** Stimulation thresholds of the Sonata gradient coil in terms of threshold gradient amplitude ΔG as a function of the rise time t for a gradient waveform with linear ramps. The thresholds are plotted as in Figure 7. The trapezoidal gradient waveform led to overall higher stimulation thresholds than the sinusoidal waveform.


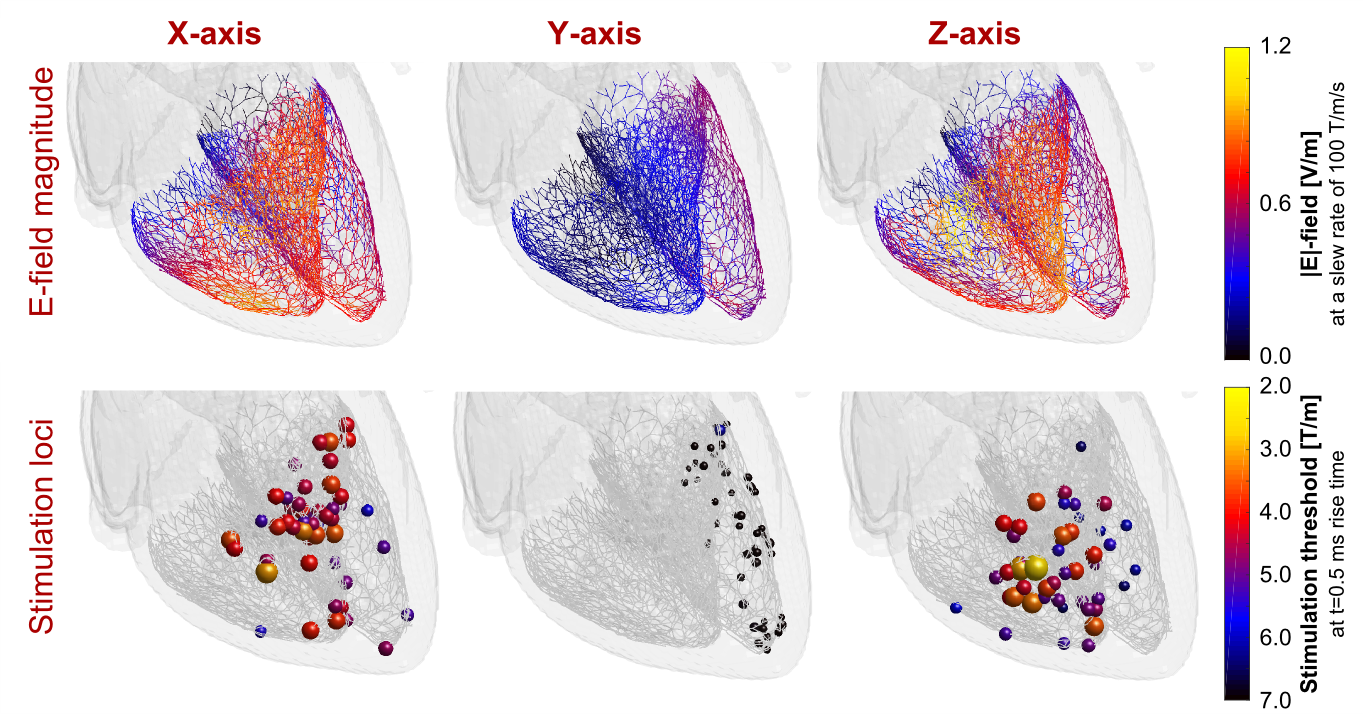


**Figure S2:** Top: E-field magnitude sampled along the Purkinje fibers of the female body model for the three gradient axes. The E-field was scaled to an equivalent slew rate of 100 T/m/s. Bottom: Stimulation loci of the 50 most sensitive fibers in the Purkinje network (larger spheres correspond to lower thresholds).


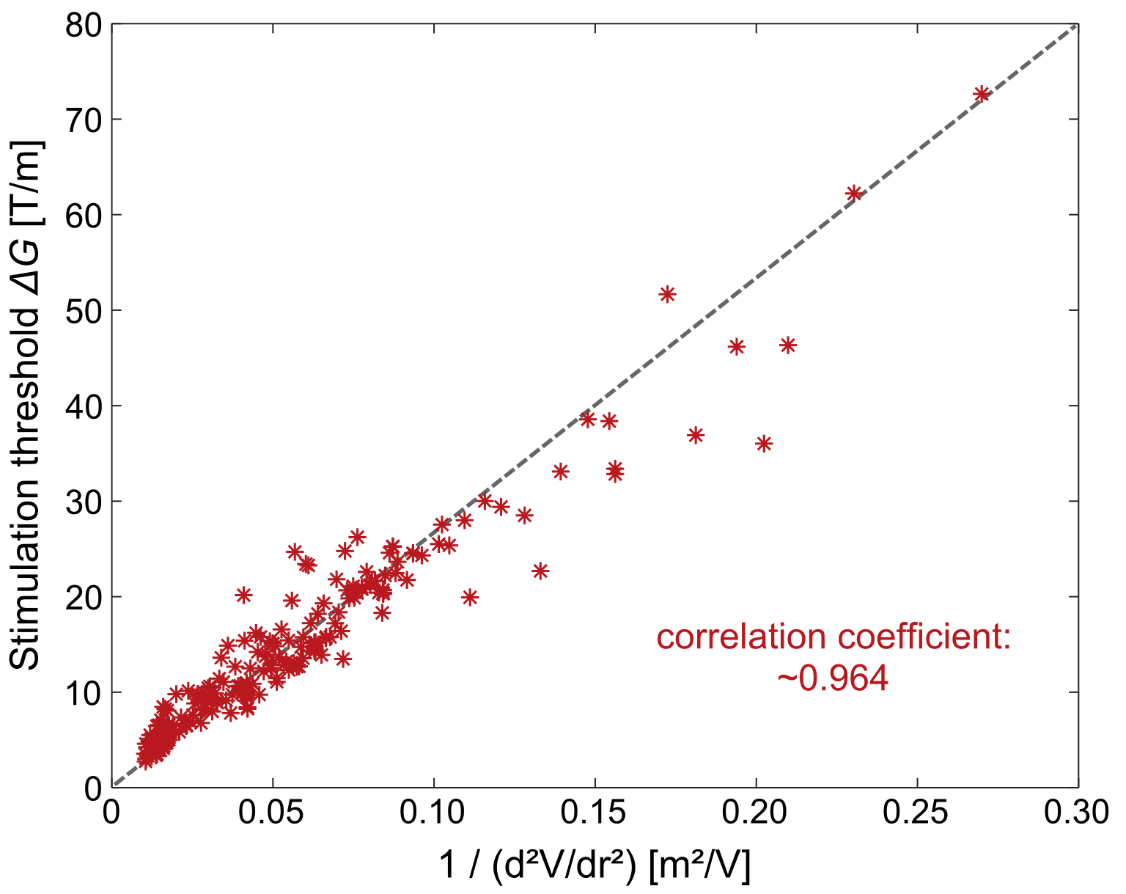


**Figure S3:** Stimulation thresholds of the Purkinje fibers (female body model, gradient Z-axis) as a function of the inverse second spatial derivative of the electric potential along the respective fiber.


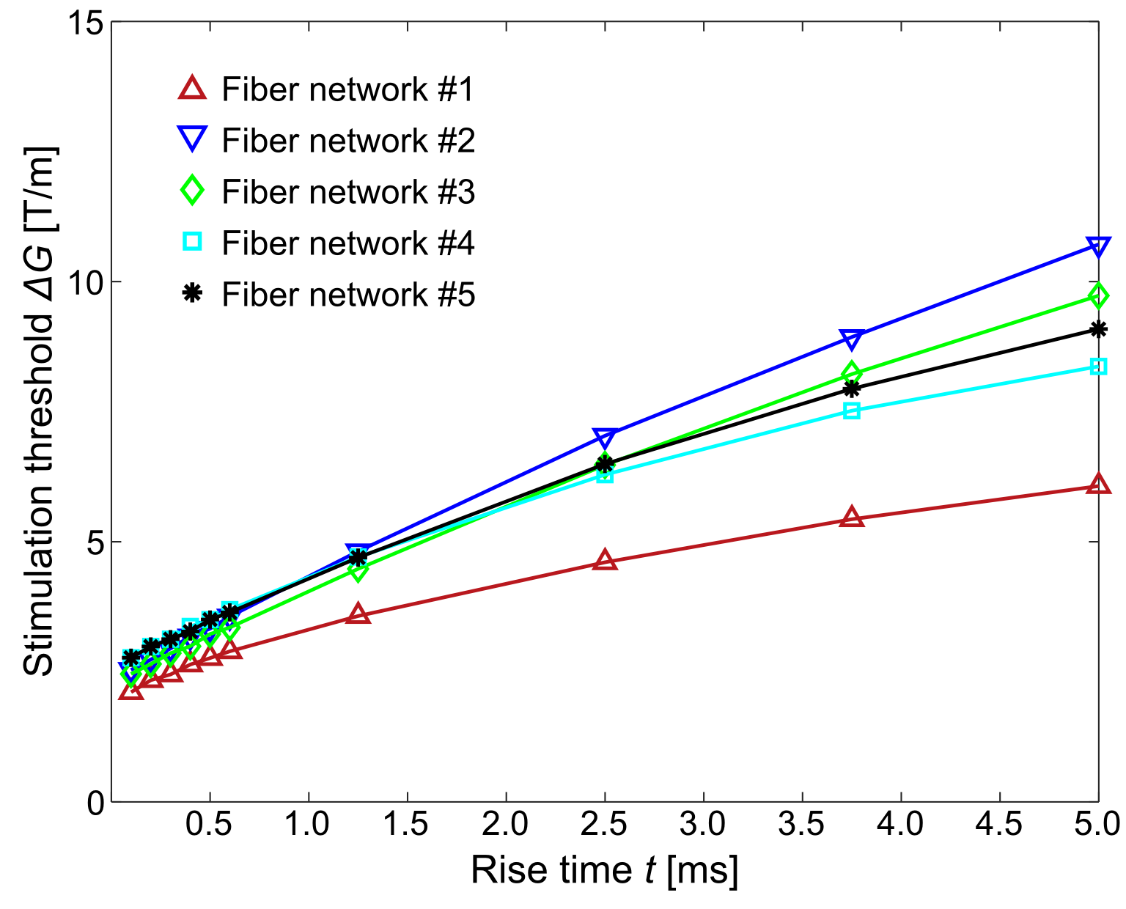


**Figure S4:** Stimulation thresholds (female body model, gradient Z-axis) computed for 5 different Purkinje fiber networks generated with the random Purkinje growth algorithm. Stimulation thresholds were modeled for a gradient waveform with sinusoidal ramps and rise times between 0.2 ms and 5.0 ms. The threshold variability across the different fiber networks ranged from 30% at short rise times up to 75% at long rise times.


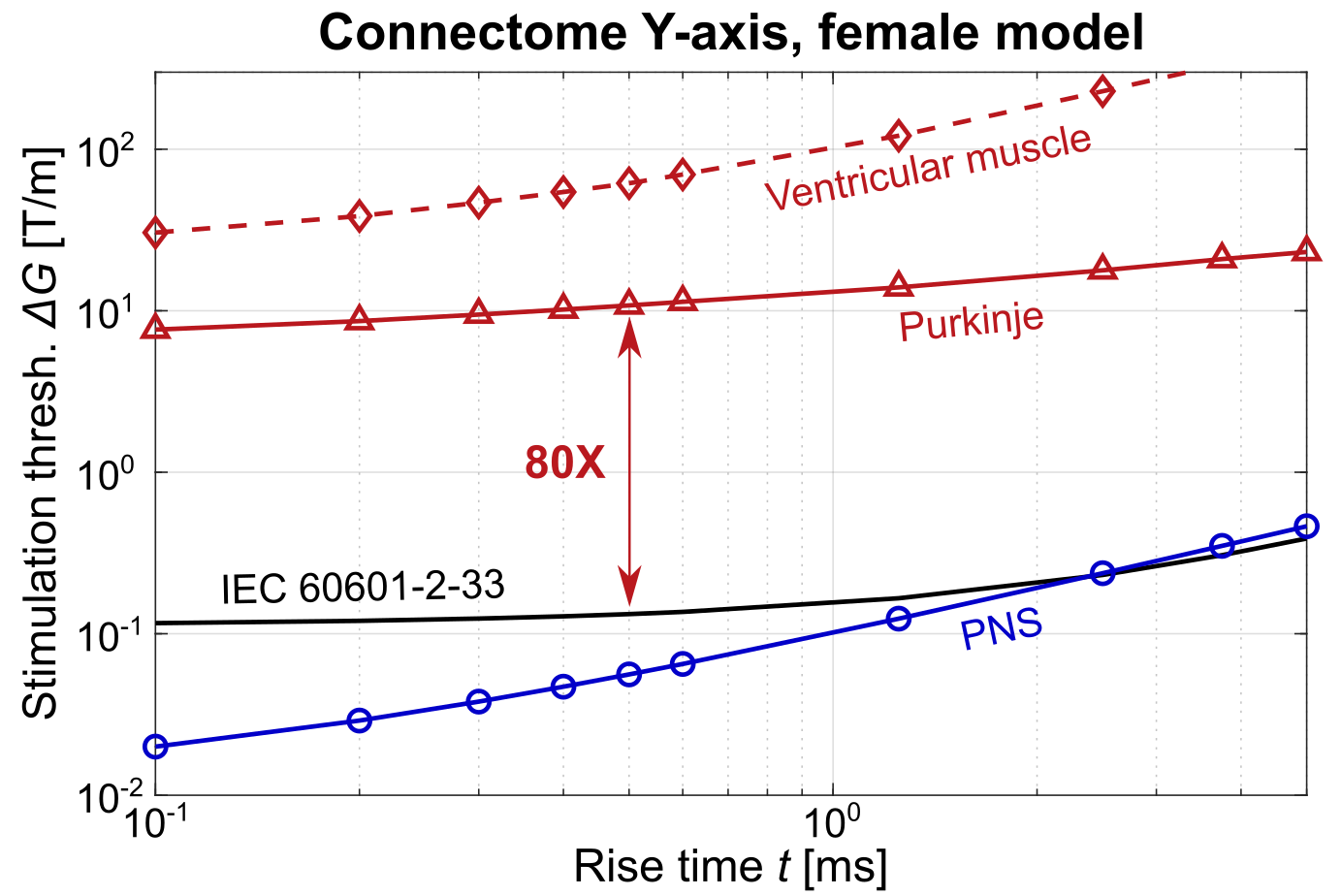


**Figure S5:** Stimulation thresholds of the MGH-UCLA Connectome gradient coil Y-axis in terms of gradient amplitude ΔG as a function of the rise time t. The CS thresholds (red) were simulated for a gradient waveform with sinusoidal ramps (500 µs flat-top duration, 10 bipolar pulses) in the female body model with the head placed at isocenter. The IEC 60601-2-33 cardiac safety limits are shown in black. The PNS thresholds (blue) were previously measured in an experimental study in healthy volunteers for rise times between 0.1 ms and 0.8 ms [14], and were extrapolated linearly for higher rise times.


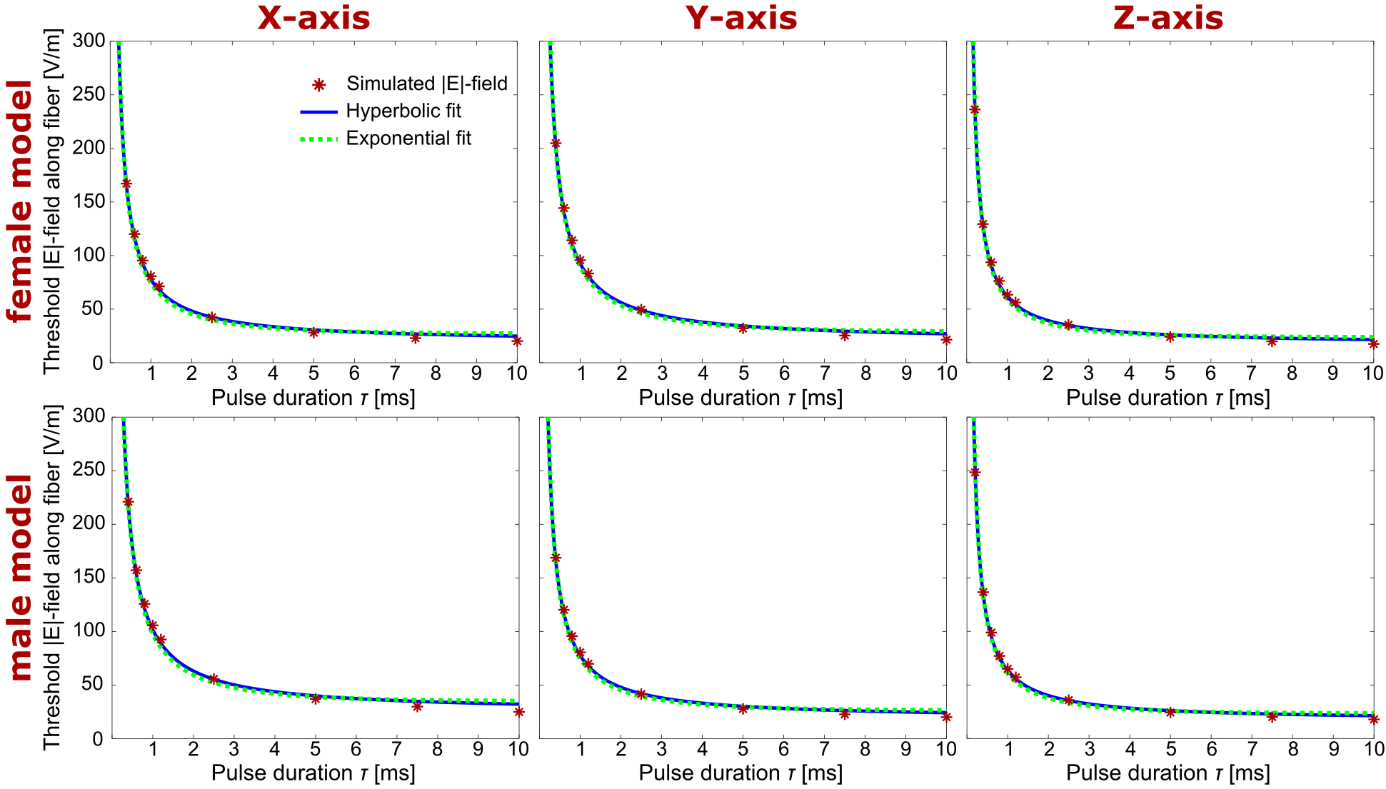


**Figure S6:** Strength-duration fit curves (hyperbolic Lapicque expression in solid blue, exponential Blair expression in dashed green) in terms of threshold E-field magnitude along the most sensitive cardiac fiber as a function of E-field pulse duration for the male and female body models (rows) and the three gradient axes (columns).


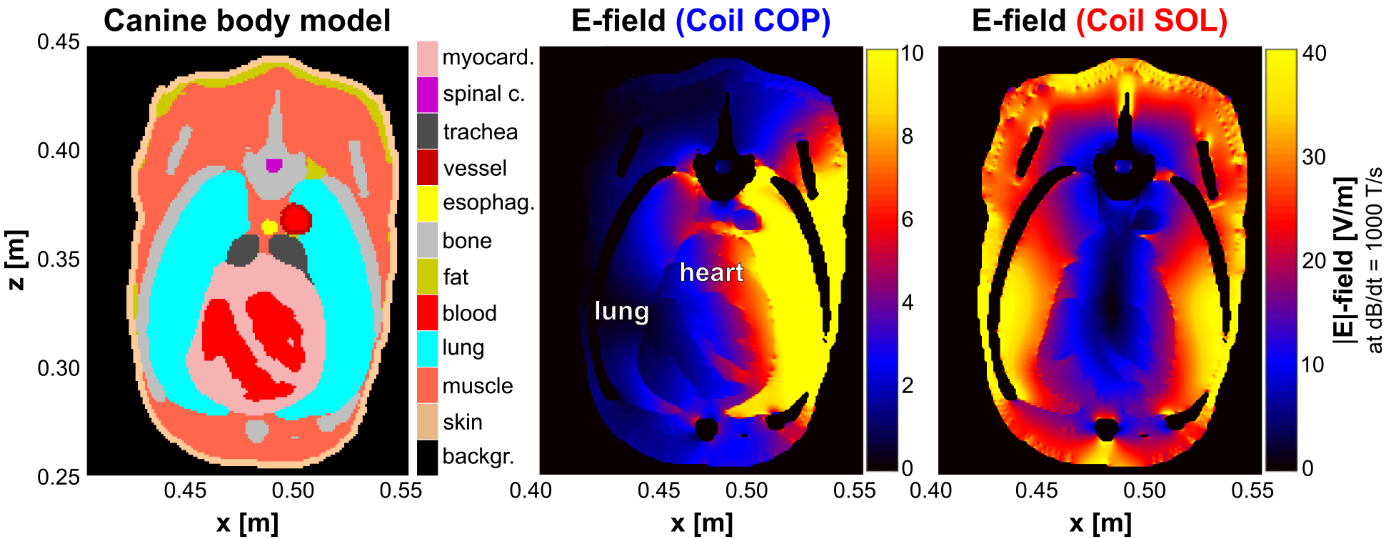


**Figure S7:** Transverse slices of the voxel model and the E-field maps induced in the 17 kg canine body model by the coplanar coils (COP) and the solenoid coil (SOL) at a peak magnetic field switching rate of dB/dt = 1000 T/s at coil center. The E-field in the bones is set to zero.
